# Urinary microbiota diversity and composition in patients with advanced renal cell cancer

**DOI:** 10.1002/bco2.70186

**Published:** 2026-05-05

**Authors:** Frederico Leal, Romualdo Morandi Filho, Lilian T. Inoue, Vitor Heidrich, Ernande X. dos Santos, Diogo A. Bastos, Anamaria A. Camargo, Denis L. F. Jardim

**Affiliations:** ^1^ Hospital Sírio‐Libanês São Paulo Brazil; ^2^ Department of Medicine State University of Campinas Campinas Brazil; ^3^ Segata Lab of Computational Metagenomics University of Trento Trento Italy; ^4^ Oncoclinicas & Co – Medica Scientia Innovation Research (MEDISIR) São Paulo Brazil

**Keywords:** biomarkers, kidney cancer, renal cell carcinoma, urinary microbiome, urinary microbiota

## Abstract

**Objectives:**

This study aims to investigate the role of urinary microbiota in renal cell carcinoma; we analysed urinary microbiota in kidney cancer patients and explored its potential role as biomarker.

**Subjects and methods:**

Samples were collected from 49 males (28 patients planned to undergo systemic therapy and 21 healthy volunteers). Two samples were collected from each patient, one prior to treatment and one after 8 to 12 weeks of systemic therapy. Microbiota was analysed by 16S rRNA sequencing. Microbiota diversity, taxonomic composition and relative abundance were compared between groups and longitudinal samples.

**Results:**

Amplicon sequence variant (ASV) richness was higher in renal cancer patients (*p* = 0.042) than controls. Beta diversity also differed between patients and controls by means of Jaccard (*p* = 0.001), Bray–Curtis (*p* = 0.008), and nonweighted UniFrac metrics (*p* = 0.001). *Acetobacter, Lacticaseibacillus, Alloscardovia, Brevibacterium* and the family Propicionibactericeae had higher relative abundance in cancer patients, while *Prevotella*, *Microbacterium* and *Sphingomonas* were more abundant in controls. Beta diversity differed between pretreatment and posttreatment samples (*p* = 0.008). After systemic treatment, we found an increased relative abundance for *Prevotella, Rothia, Bradyrhizobium, Methylobacterium/Methylobrum, Porphiromonas* and *Fusobacterium* and a decreased one for the *Burkeholderia‐Caballeronia‐Paraburkholderia* group. Higher ASV richness was predictive of poor prognosis for RCC patients (*p* = 0.043) but not of treatment response.

**Conclusions:**

Urinary microbiota in patients with renal cell carcinoma differed from controls. Changes in microbiota composition were observed after systemic treatment. Urinary microbiota should be further investigated as a potential biomarker in renal cell carcinoma.

## 1 INTRODUCTION

Alterations in microbiota diversity and composition have been investigated in several malignancies,^1^, ^2^ including renal cell carcinoma (RCC). Imbalances in gut microbiota have been implicated in host anticancer immune responses, leading to carcinogenicity and modulation of treatment responses.^3^

Studies have shown that faecal microbiota composition may predict response to targeted therapy and immunotherapy in patients with advanced RCC.^4^, ^5^ Moreover, microbiota modification by antibiotic therapy has been shown to affect immunotherapy treatment response.^6^ Finally, clinical trials have reported that interventions in gut microbiota may improve outcomes.^7^

The role of urinary microbiota in genitourinary tumours is much less understood. Until recently, healthy urine was considered sterile, but novel metagenomic analyses have shown that human urine actually harbours a rich microbiome, and its microbiota can be a potential biomarker in several diseases, including genitourinary cancers.^8^, ^9^

Recent studies have shown that urinary microbiota in bladder cancer patients significantly differs from healthy volunteers and have also reported differences in treatment response according to microbiota composition.^8^, ^10^, ^11^ However, studies on urinary microbiota in RCC are still lacking. Unlike bladder cancer, RCC cells are not in direct contact with urine, especially in patients previously treated with radical nephrectomy, but, as described above, interaction between neoplasms and microbiomes is complex and does not always require direct colonization.^1^ Gu microbiota, for instance, has been shown to impact prognosis and treatment response for RCC, despite the said neoplasms being far removed from intestinal microbiome.^3^, ^4^ Our aim was to compare urinary microbiota between healthy male volunteers and patients with advanced RCC, and its relationship with clinical behaviour of the disease and outcomes during standard of care systemic therapies.

## 2 METHODS

### 2.1 Study design

This was a prospective, noninterventional cohort study conducted in two hospitals in Brazil (Hospital Sirio‐Libanês and Hospital de Clinicas da Unicamp). Patients and volunteers were divided into three cohorts. Cohort A consisted of healthy male volunteers with no personal history of cancer. Cohort B consisted of male patients with advanced RCC for whom antiangiogenic targeted therapy was planned, while Cohort C included male patients for whom planned treatment included immunotherapy, either alone or in combination with targeted therapy.

One single urinary sample was collected from subjects in Cohort A. For Cohorts B and C, two samples were collected: one prior to the start of systemic treatment and the other from 8 to 12 weeks after therapy initiation. Urinary microbiota composition from Cohort A (Controls) was compared to the one from Cohorts B and C pretreatment samples (RCC patients). Additionally, posttreatment microbiota from Cohorts B and C were compared to pretreatment compositions.

### 2.2 Patients included

Only males were included in this study, to minimize bias related to sex‐specific microbiota differences and avoid contamination from vaginal microbiota.^12^ Patients in Cohorts B and C were required to have histologically confirmed advanced RCC and planned to start systemic treatment with targeted therapy, immunotherapy or a combination thereof. Previous systemic therapy was not allowed, but surgery and/or radiation was permitted. Subjects in Cohort A should have no personal history of cancer and no history of urological disease.

Patients with previous neoplasms (except for nonmelanoma skin cancer), inflammatory bowel disease, current or recent (less than 4 weeks from resolution) urinary tract infection, current or recent (less than 4 weeks after completion) antibiotic use and patients who refused to sign informed consent were excluded.

To ensure demographic matching between cohorts, recruitment for Cohort A commenced only after 80% of planned enrollment for Cohorts B and C was completed. Healthy volunteers' age was controlled to match the age range of the other two cohorts.

### 2.3 Sample collecting and processing

Urine samples were collected by the clean‐catch method. While urine sampling is subject to contamination, Decontam analysis (as described below) may diminish contamination risks.^13^ Immediately after collecting, samples were frozen at −80°C. Total DNA was extracted from urine samples and negative controls (1 ml phosphate‐buffered saline) using the QIAamp DNA Microbiome kit (Qiagen). One nanogram (for samples with ≥ 0.25 ng/μL) or 4 μL (for samples with < 0.25 ng/μL) of DNA was used to prepare the V1V2 amplicon libraries using the QIAseq 16S/ITS Region Panel kit (Qiagen), with the optimized cycling conditions previously described.^14^ 16S Ribosomal RNA (16S rRNA) gene hypervariable regions V1 and V2 were chosen for sequencing due to their sensitivity and specificity for taxonomic classification down to genus level in urine samples.^14^

Libraries were quantified using the QIAseq library quant assay kit (Qiagen), normalized to 2 nM and sequenced using the MiSeq Reagent Kit v3 (600‐cycles) (Illumina), following the 2 × 276 bp paired‐end read protocol.

### 2.4 Bioinformatic analyses

Paired‐end reads were demultiplexed and adapters removed. Remaining data was processed using QIIME 2 software and primers were removed by Cutadapt plugin.^15^ The sequences were denoised and truncated at length 257 to maintain a Phred quality score above 20 using the DADA2 plugin. Amplicon sequence variants (ASVs) were assigned using a naive bayes classifier trained by the Scikit plugin. Chimerical ASVs were removed by the UCHIME algorithm with the VSEARCH tool based on the SILVA microbial rRNA database.^16^ Nonbacterial ASVs were removed using VSEARCH^1^ (via q2‐vsearch) and the SILVA database (v138.1). Following the taxonomic assignment of ASVs, nonbacterial and contaminant ASVs were filtered out (via q2‐taxa filter‐seqs). Finally, additional contaminant ASVs were removed with Decontam (via q2‐quality‐control Decontam), based on the prevalence of ASVs detected in the four negative technical controls (sterile PBS). QIIME 2 outputs were analysed in the R environment.^15^

### 2.5 Statistical Analysis

Data were normalized by scaling with ranked subsampling (SRS) at 476 reads per sample.^15^ Diversity estimates were conducted with normalized data.

Alpha diversity was computed using ASV richness, Shannon, Gini‐Simpson and Faith phylogenetic distance indexes.^17^, ^18^ Wilcoxon rank sum test was performed to compare alpha diversity obtained from different samples.

Beta diversity was measured by Jaccard, Bray–Curtis and weighted and nonweighted UniFrac metrics^17^ and analysed with the PERMANOVA test. Compositions were visualized by Principal Coordinate Analysis (PCA).

Taxa relative abundance was also analysed. Differential abundance was analysed with ANCOM‐BC2, with multiple test correction using the Holm method.^15^

## 3 RESULTS

### 3.1 Patients Included

From September 2020 to October 2023, we recruited 28 RCC patients and 21 healthy controls (Cohort A). Among RCC patients, 17 were treated with targeted therapy alone (Cohort B), and 11 received immunotherapy, either alone or in combination with targeted therapy (Cohort C). Twenty‐two patients had previous treatment with radical nephrectomy, while one was submitted to nephrectomy after systemic treatment (and after collection of both urinary samples), and five patients were not treated with nephrectomy.

Median age was 61.2 (42.6–80.4) years and was similar between cohorts. Eight RCC patients were IMDC favourable risk, 16 were intermediate risk and 5 were poor risk. About 86.2% of patients had clear cell RCC. Data on the included population are summarized in Table 1.

**TABLE 1:** Patient characteristics.

Cohort	Healthy	Renal cell carcinoma		Total
	A	B	C	
Number	21	18	11	50
Age				
Median	59.5	60.8	66.6	61.2
Minimum	42.6	45.2	44.3	42.6
Maximum	72.5	80.4	74.7	80.4
Ethnicity (%)				
White	85.7	72.2	90.9	82
Other	14.3	27.8	9.1	18
Histology (%)				
Clear cell	NA	83.3	91	86.2
Other cells	NA	16.7	9	13.8
IMDC, *n* (%)				
Favourable	NA	4 (16.6)	4 (36.4)	8 (27.6)
Intermediate	NA	10 (41.6)	6 (54.5)	16 (55.2)
Poor	NA	4 (16.6)	1 (9.1)	5 (17.2)
Treatment, *n*				
Pazopanib	NA	18	0	18
Ipilimumab + nivolumab	NA	0	8	8
Pembrolizumab + axitinib	NA	0	3	3
Best response, *n*				
Complete response	NA	0	2	2
Partial response	NA	3	6	9
Stable disease	NA	5	3	8
Progressive disease	NA	5	0	5
Death	NA	3	0	4
Lost follow‐up	NA	1	0	1

Median progression free survival (PFS) for the entire population of RCC patients (cohorts B plus C) was 11.7 months. Median PFS was higher in Cohort C (24.1 months) than Cohort B (7.4 months). According to IMDC group risk stratification, median PFS was 16.4, 15.1 and 2.5 months for favourable, intermediate and poor risk, respectively.

### 3.2 Urinary microbiota from RCC patients differed significantly from controls

Libraries had a minimum of 123 000 reads and an average of 240 000 reads for analysis. Collected samples were compared to negative controls, and contaminant ASVs were removed.

**FIGURE 1:**
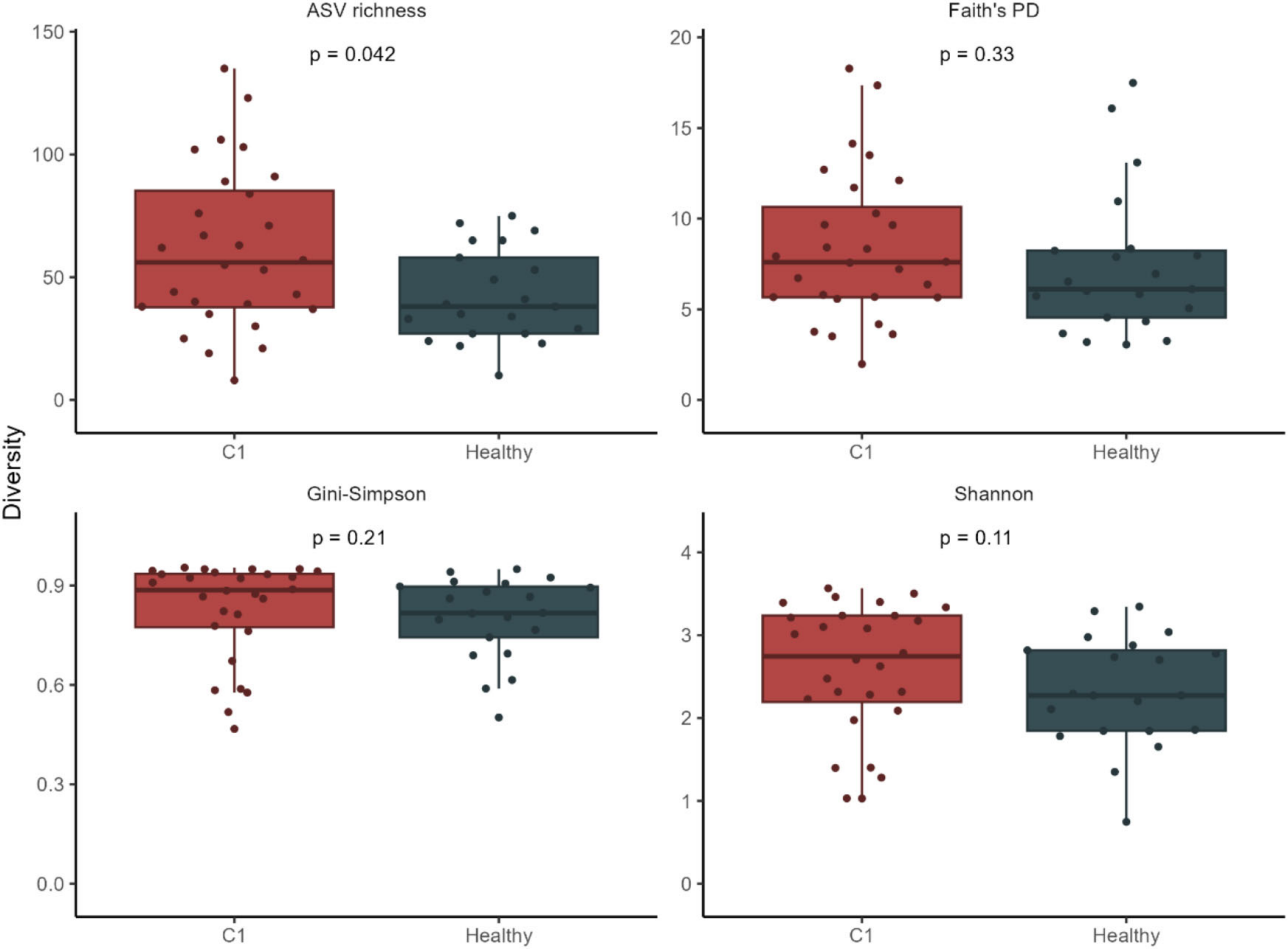
Alpha diversity comparisons between pretreatment RCC patients (red, *n* = 28) and healthy controls (blue, *n* = 21). Wilcoxon ranksum test was used.

ASV richness was higher in samples from RCC patients when compared to healthy volunteers (*p* = 0.042). No statistically significant difference was observed between patients and controls for other alpha diversity indexes. (Figure 1).

**FIGURE 2:**
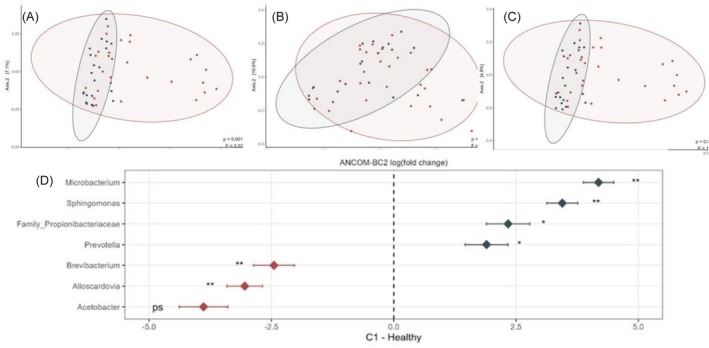
(A) Beta diversity comparison between pretreatment RCC samples (red) and controls (blue) using the Jaccard. (B) Bray–Curtis. (C) Nonweighted UniFrac metrics. PERMANOVA test was used. (D) ANCOMBC2 analysis comparing pretreatment samples from RCC patients versus samples from healthy controls. Genera with relative abundances higher in RCC patients are shown in red (left side of dotted line); ASVs with higher relative abundance in controls are shown in blue (right side of dotted line. * Represents results with *p* < 0.05; ** for results with *p* < 0,001, ps for pass on sensitivity test).

Beta diversity analysis showed differences in microbiota composition between RCC patients and controls according to Jaccard (Figure 2A), Bray–Curtis (Figure 2B) and nonweighted UniFrac metrics (Figure 2C). No difference was observed in the weighted UniFrac metric (see Figure S1 for details).

Some bacterial genera had higher relative abundance in RCC patients (*Acetobacter, Alloscardovia* and *Brevibacterium*), while *Microbacterium*, *Sphingomonas* and genera from the family Propicionibactericeae were more abundant in healthy controls (see Figure 2D).

### 3.3 Urinary microbiota may be prognostic but does not correlate with IMDC

ASV richness was marginally predictive of poor prognosis in RCC patients treated with antiangiogenic targeted therapies (Cohort B). Patients with ASV richness index above the median presented inferior PFS when compared to below median richness patients when treated with antiangiogenic targeted therapy (See Figure S2). However, no prognostic impact was observed in the overall RCC population (regardless of treatment strategy) (see Figures S3–S5). The number of events in patients receiving immunotherapy (Cohort C) was too small for Kaplan–Meier analysis. No other alpha diversity metrics were predictive of PFS.

Alpha diversity indexes did not differ between IMDC group risks. Also, no significant difference in beta‐diversity was observed between IMDC risk groups (see Figures S6 and S7 for details).

### 3.4 Microbiota composition was modified by systemic treatment but was not predictive of response

**FIGURE 3:**
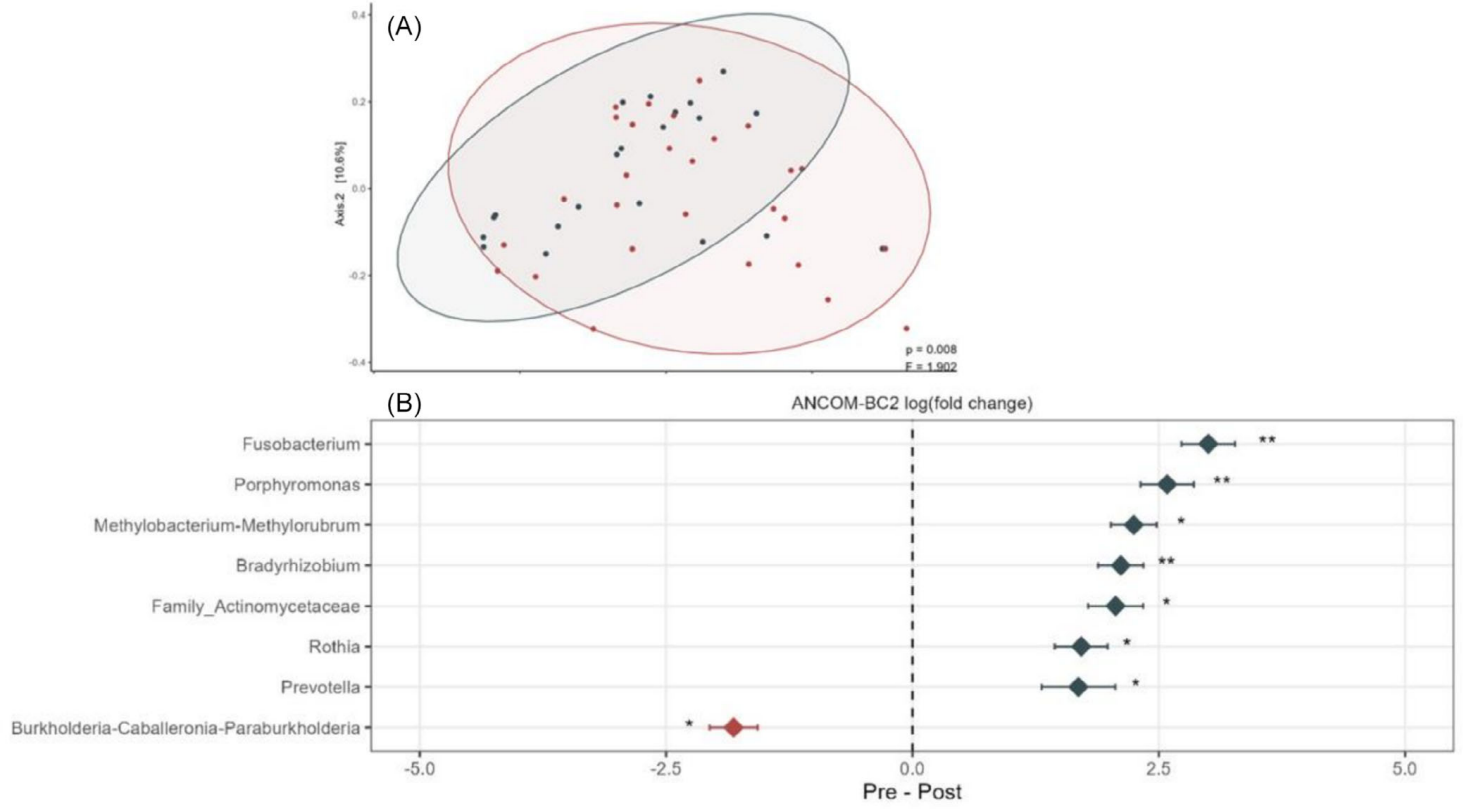
(A) Beta diversity comparison between pretreatment RCC samples (red) and posttreatment RCC samples (blue) using the nonweighted Unifrac metric. PERMANOVA test was used. (B) ANCOMBC2 analysis comparing pretreatment versus posttreatment samples from RCC patients. Genera with relative abundances higher pretreatment are shown in red (left side of dotted line), while genera with higher relative abundance posttreatment are shown in blue (right side of dotted line).

Pretreatment and posttreatment samples differed in microbiota composition according to the nonweighted UniFrac metric (Figure 3A) but not with Jaccard, Bray–Curtis or weighted UniFrac metrics. There were no significant differences in alpha diversity between pretreatment and posttreatment samples (see Figure S8).

Relative abundance of some bacterial genera (*Prevotella, Rothia, Bradyrhizobium, Methylobacterium/Methylobrum, Porphiromonas* and *Fusobacterium*) was higher after 2–3 months of antineoplastic systemic treatment when compared to pretreatment RCC patients' samples (Figure 3B). On the other hand, genera from the *Burkeholderia‐Caballeronia‐Paraburkholderia* group had lower relative abundance after systemic treatment (Figure 3B).

There were no statistically significant differences in alpha or beta diversity between responders and nonresponders to treatment, either in Cohorts B or C. No ASV relative abundance was found to be predictive of response.

## 4 DISCUSSION

Our study demonstrated differences between urinary microbiota from RCC patients when compared to healthy controls, including differences in alpha diversity, beta diversity and relative abundance of specific bacterial genera. We also found that current systemic treatment administration for advanced RCC is able to modify urinary microbiota composition. Finally, we found that higher urinary microbiota diversity was marginally associated with poor prognosis in advanced RCC, though only when measured by ASV richness index. To our knowledge, this is the first study to evaluate urinary microbiota in advanced RCC patients undergoing systemic therapy.

*Acetobacter, Alloscardavia* and *Brevibacterium* were more abundant in pretreatment RCC patients when compared to controls. Genus *Acetobacter* is known for acetic acid synthesis and therefore may modulate availability of nutrients for other microorganisms, further modifying urinary microbiota.^19^ Genera *Alloscardavia* and *Brevibacterium* are commonly found in urinary microbiota, though their clinical significance is not yet established.^18^, ^20^ We found genera *Microbacterium*, *Sphingomonas* and *Prevotella* to have higher relative abundance in controls. Genus *Prevotella* is associated with urinary tract infections in females, though its role in male urinary microbiome is not known.^21^ Interestingly, both *Sphingomonas* and *Microbacterium* ^22^ are potentially pathogenic genera, with some infections described in medical literature, and *Sphingomonas* has even been associated with bladder cancer risk,^23^ but their relative abundance was higher in controls in our study. Moreover, studies in bladder cancer have found significant differences in urinary microbiota between patients and controls, albeit their results are somewhat inconsistent. Some studies have reported higher alpha diversity in samples from cancer patients,^24^ while others found no significant differences in diversity indexes.^11^, ^25^ All studies have, however, found some taxa have different relative abundance between groups. Our study shows that urinary microbiome in RCC is different from controls, consistent with findings reported for other genitourinary neoplasms.^9^, ^24^ Ahn and colleagues have also reported significant differences in microbiota composition between RCC patients and controls, but the bacterial genera found to differ (*Cutibacterium acnes*, *Cutibacterium granulosum*, *Peptoniphilus lacydonensis*, and *Tessaracoccus*) were distinct from the ones in our study.^9^

In contrast to previous studies on faecal microbiota, our analyses did not find urinary microbiota to be predictive of treatment response in RCC.^4^ This result may be limited by the small sample size in our study. However, our analyses did find statistically significant differences between pretreatment and posttreatment urinary microbiota. Beta diversity, as measured by nonweighted UniFrac metric, showed differential microbiota composition. Genera *Prevotella, Rothia, Bradyrhizobium, Methylobacterium/Methylobrum, Porphiromonas* and *Fusobacterium* had their relative abundance increased after treatment initiation. As described above, *Prevotella* relative abundance was higher in controls than in RCC, so this increase with treatment in RCC patients is noteworthy. Genus *Rothia* is considered a normal component of human microbiomes.^18^ *Fusobacterium*, *Methylobacterium* and *Methylobrum* are opportunistic germs occasionally associated with infection^26^ and have also been associated with bladder cancer.^27^ *Bradyrhizobium* and *Porphiromonas* have been reported to exhibit higher abundance in recurrent bladder cancer.^28^, ^29^ Interestingly, our group also analysed the urinary microbiota composition in bladder cancer patients pre‐intravesical and postintravesical BCG and did not find significant alterations in diversity nor composition between samples^8^ though this paper by James and collegues^30^ reports differences between pretreatment and posttreatment microbiota composition in bladder cancer and, interestingly, also found some bacterial genera to be predictive of response. We also found inferior PFS in patients undergoing antiangiogenic therapy which had above‐median ASV richness compared to ones with below‐median richness index. While such finding must be considered preliminary due to our small sample size and the multiple metrics analysed, it interestingly contrasts with studies which evaluated faecal microbiome, for which patients with higher richness index have been reported to present improved prognosis.^31^ Therefore, urinary microbiota may be prognostic for advanced RCC, albeit in a different fashion from gut microbiota. It is important to note that the possible prognostic implication of microbiota composition may be independent of clinical factors, as no correlation was noted between IMDC and our microbiota analysis.

Our study has some limitations. First, to avoid contamination from vaginal microbiota,^12^ we recruited male participants only. Hence, our samples may not be representative of all RCC patients, including females. Also, 16S rRNA V1 and V2 analysis is highly specific for bacterial taxa down to genus level but not so much for species.^14^ Therefore, it is possible that some bacterial species within the same genus may be differentially associated with RCC, and such differences could not be detected by our analyses. Finally, given our small sample size, some significant associations may have been missed as a result of limited statistical power. Therefore, our findings should be replicated in larger studies to further establish the role of urinary microbiota as a biomarker in RCC.

## 5 CONCLUSION

Urinary microbiota in RCC patients was found to be significantly different from healthy controls, including differences in alpha diversity, beta diversity and differences in genera relative abundance. Significant differences in microbiota composition were observed during systemic treatment. Urinary microbiota diversity and composition were not predictive of treatment response, but higher ASV richness was predictive of poor survival in RCC patients treated with targeted therapy. Further research on urinary microbiota as a biomarker in RCC is warranted.

## AUTHOR CONTRIBUTIONS

*Study concept, study design, samples collection and manuscript preparation*: Frederico Leal. *Data processing, data analysis and statistical analysis*: Romualdo Morandi Filho. *Sample preparation and manuscript review*: Lilian T. Inoue. *Data analysis and statistical analysis*: Vitor Heidrich. *Samples collection and sample preparation*: Ernande X. dos Santos. *Study design and manuscript review*: Diogo A. Bastos. *Study concept, study design, data analysis and manuscript review*: Anamaria A. Camargo. *Study concept, study design, samples collection and manuscript review*: Denis L. F. Jardim.

## CONFLICT OF INTEREST STATEMENT

The authors certify that there is no conflict of interest with any financial organization regarding the material discussed in the manuscript.

## Supporting information


**Figure S1:** Weighted UniFrac metrics comparing urinary microbiota composition from RCC patients (Red) to healthy controls (blue).

**Figure S2:** Kaplan–Meier progression‐free survival curves comparison for RCC patients with higher (Red) versus lower ASV richness (Blue). (A) Patients treated with antiangiogenic targeted therapy. (B) overall RCC patients.

**Figure S3:** Alpha diversity comparisons nonresponders (red) and responders to treatment (blue). Wilcoxon ranksum test was used.

**Figure S4:** (A) Beta diversity comparison between nonresponders to antiangiogenic targeted therapy (red) and responders (blue) using the Jaccard. (B) Bray–Curtis. (C) nonweighted UniFrac. (D) weighted UniFrac metrics. PERMANOVA test was used.

**Figure S5:** (A) Beta diversity comparison between nonresponders immunotherapy (red) and responders (blue) using the Jaccard. (B) Bray–Curtis. (C) Nonweighted UniFrac. (D) weighted UniFrac metrics. PERMANOVA test was used.

**Figure S6:** Alpha diversity comparisons between favourable risk IMDC RCC patients (Red, *n* = 8) and intermediate plus poor risk IMDC patients (Blue, *n* = 21). Wilcoxon ranksum test was used.

**Figure S7:** (A) Beta diversity comparison between favourable risk IMDC RCC patients (Red) and intermediate plus poor risk IMDC patients (Blue) using the Jaccard. (B) Bray–Curtis. (C) Nonweighted UniFrac. (D) weighted UniFrac metrics. PERMANOVA test was used.

**Figure S8:** (A) Beta diversity comparison between pretreatment RCC samples (Red) posttreatment samples (Blue) using the Jaccard. (B) Bray–Curtis. (C) weighted UniFrac metrics. PERMANOVA test was used.
