# Supplementary material for: Urinary microbiota diversity and composition in patients with advanced renal cell cancer
Source: BJUI Compass. 2026 May 5;7(5):e70186. doi: 10.1002/bco2.70186 (PMC13143510; doi:10.1002/bco2.70186)
Supplement: Supplementary file 1 — Figure S1: Weighted UniFrac metrics comparing urinary microbiota composition from RCC patients (Red) to healthy controls (blue). [file BCO2-7-e70186-s005.docx]

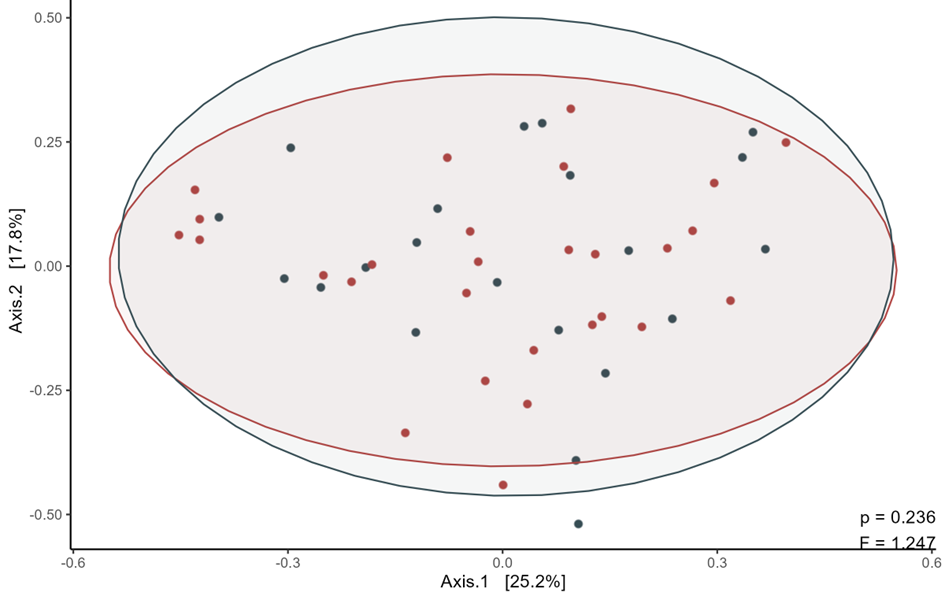


Supplemental Figure 1: Weighted UniFrac metrics comparing urinary microbiota composition from RCC patients (Red) to healthy controls (blue)
