# Supplementary material for: Urinary microbiota diversity and composition in patients with advanced renal cell cancer
Source: BJUI Compass. 2026 May 5;7(5):e70186. doi: 10.1002/bco2.70186 (PMC13143510; doi:10.1002/bco2.70186)
Supplement: Supplementary file 2 — Figure S2: Kaplan–Meier progression‐free survival curves comparison for RCC patients with higher (Red) versus lower ASV richness (Blue). (A) Patients treated with antiangiogenic targeted therapy. (B) overall RCC patients. [file BCO2-7-e70186-s001.docx]

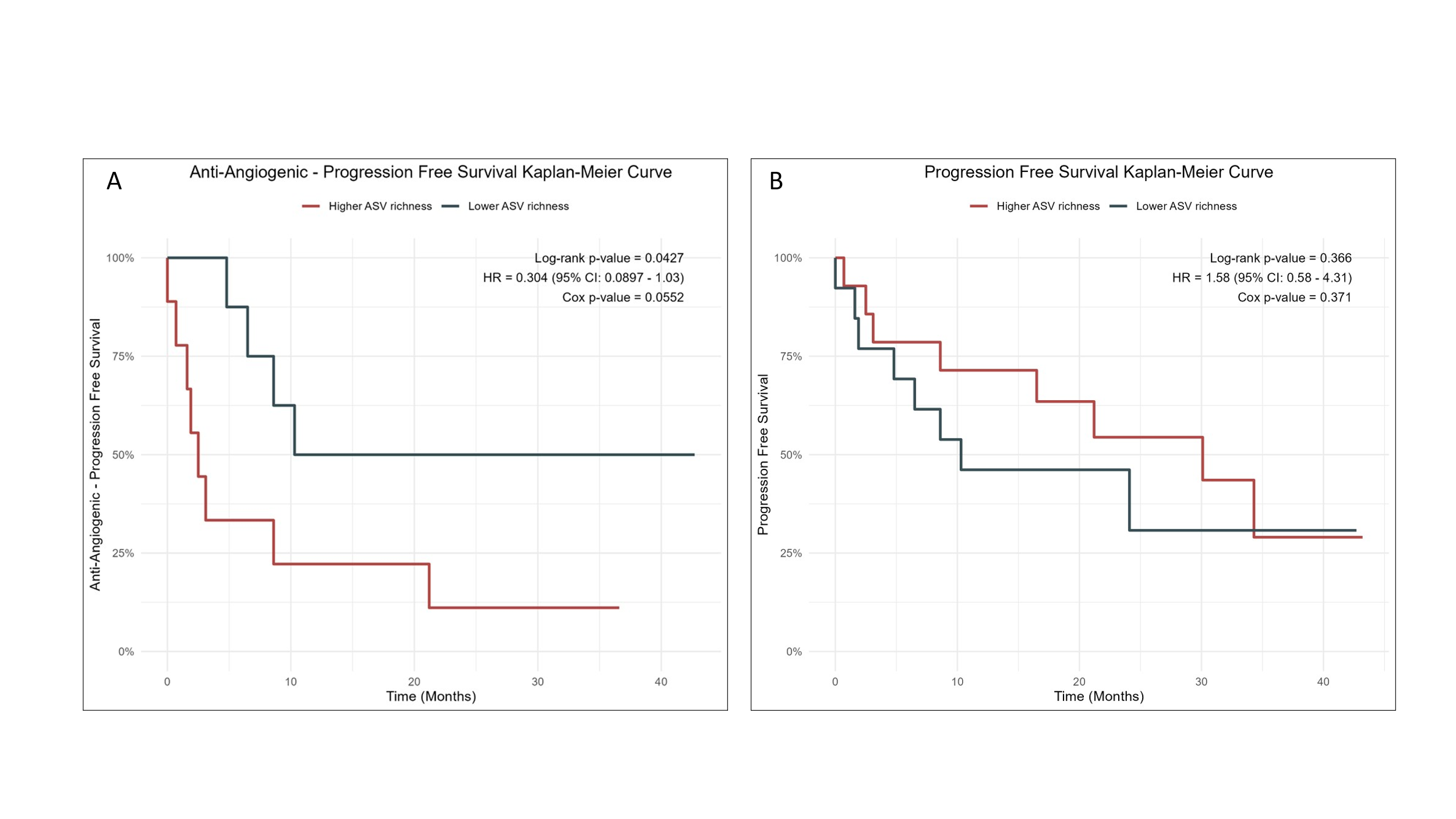


*Supplemental Figure 2: Kaplan-Meier progression-free survival curves comparison for RCC patients with higher (Red) versus lower ASV richness (Blue) .* ***A:*** *Patients treated with antiangiogenic targeted therapy* ***B:*** *overall RCC patients.*
