# Supplementary material for: Urinary microbiota diversity and composition in patients with advanced renal cell cancer
Source: BJUI Compass. 2026 May 5;7(5):e70186. doi: 10.1002/bco2.70186 (PMC13143510; doi:10.1002/bco2.70186)
Supplement: Supplementary file 3 — Figure S3: Alpha diversity comparisons nonresponders (red) and responders to treatment (blue). Wilcoxon ranksum test was used. [file BCO2-7-e70186-s002.docx]

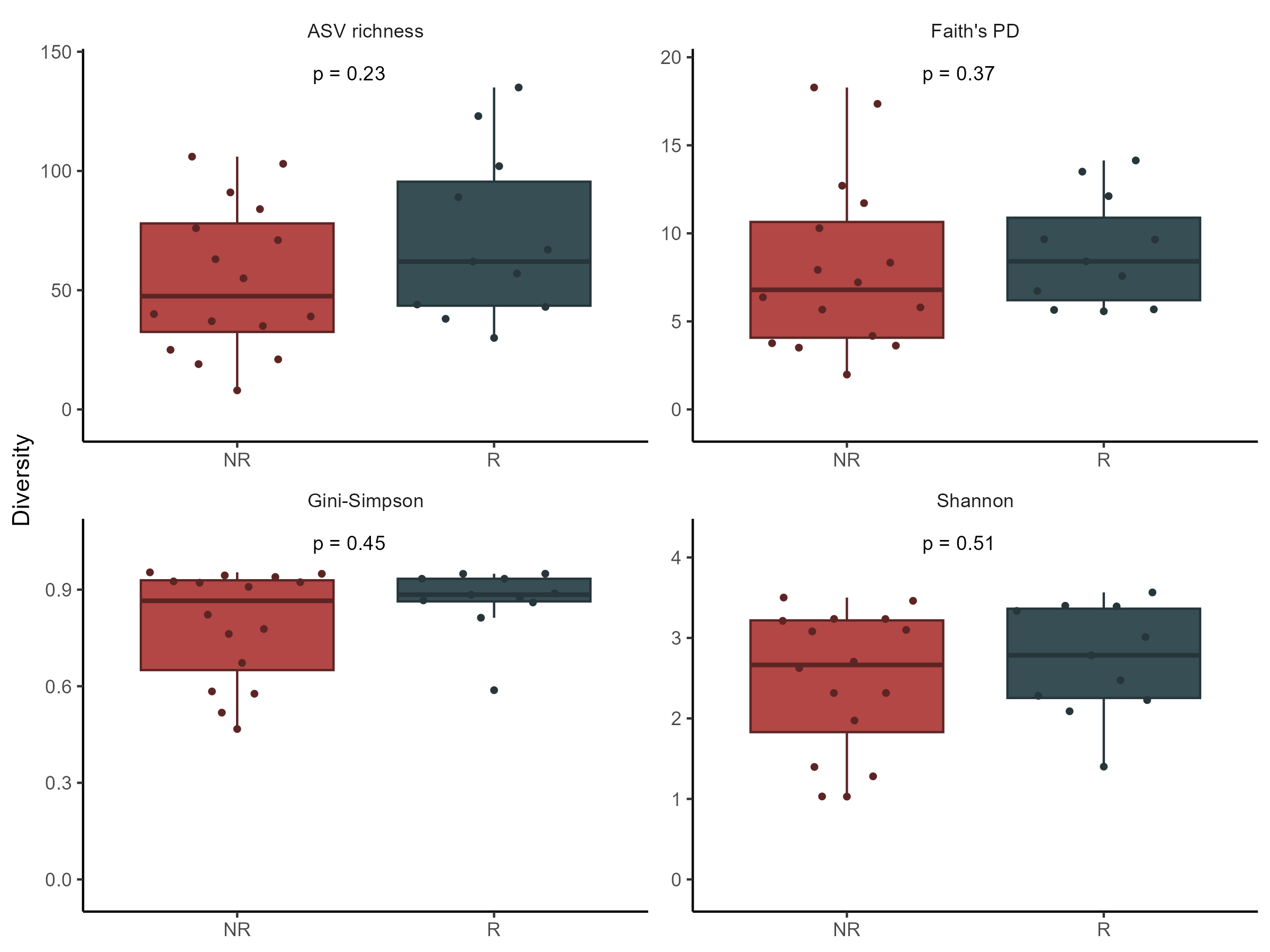


Supplemental Figure 3: Alpha diversity comparisons non-responders (Red) and responders to treatment (Blue). Wilcoxon ranksum test was used
