# Supplementary material for: Urinary microbiota diversity and composition in patients with advanced renal cell cancer
Source: BJUI Compass. 2026 May 5;7(5):e70186. doi: 10.1002/bco2.70186 (PMC13143510; doi:10.1002/bco2.70186)
Supplement: Supplementary file 5 — Figure S5: (A) Beta diversity comparison between nonresponders immunotherapy (red) and responders (blue) using the Jaccard. (B) Bray–Curtis. (C) Nonweighted UniFrac. (D) weighted UniFrac metrics. PERMANOVA test was used. [file BCO2-7-e70186-s007.docx]

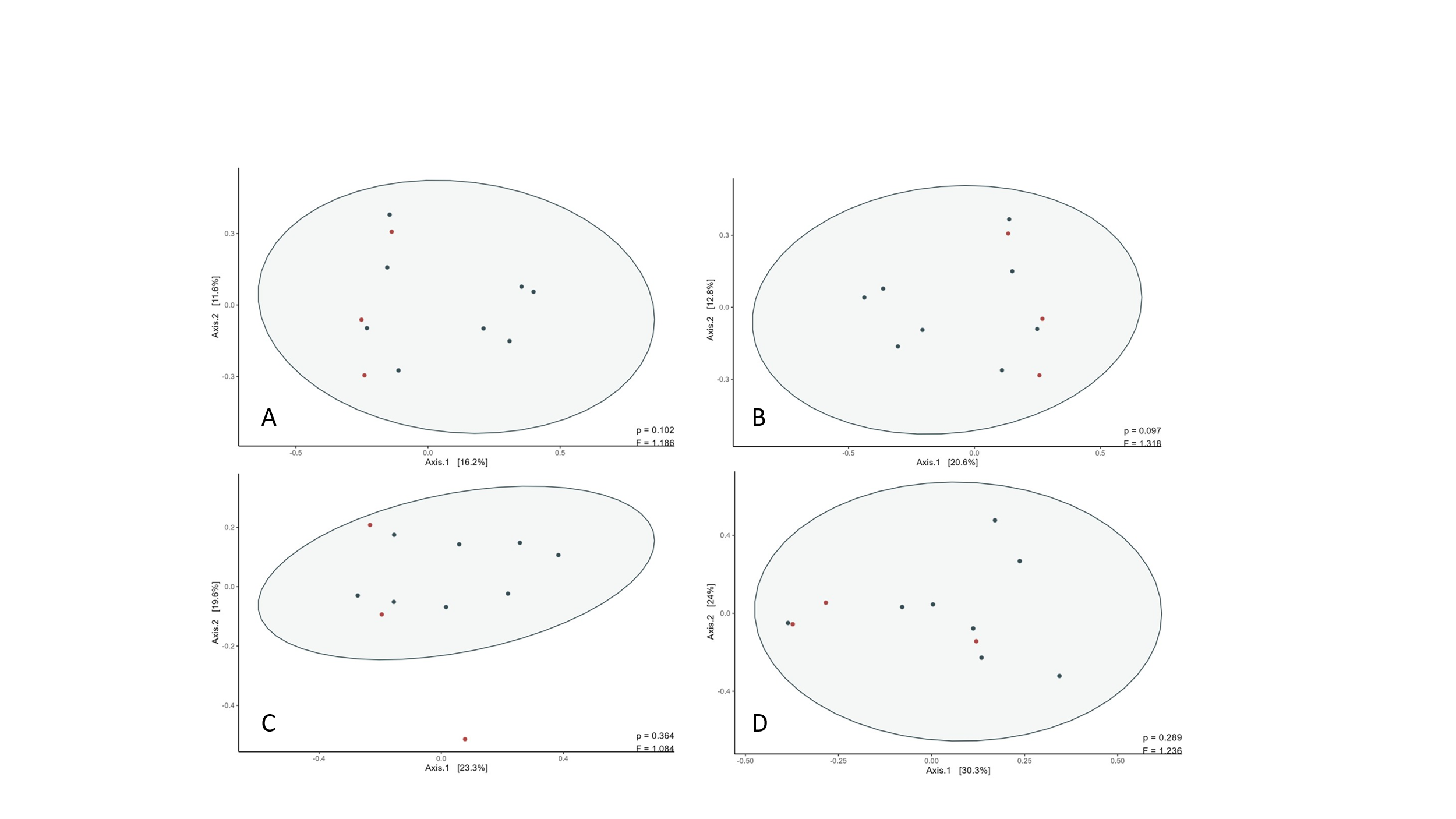


Supplemental Figure 5: A: Beta diversity comparison between non-responders immunotherapy (Red) and responders (Blue) using the Jaccard B: Bray-Curtis C: non-weighted UniFrac D: weighted UniFrac metrics. PERMANOVA test was used.
