# Supplementary material for: Urinary microbiota diversity and composition in patients with advanced renal cell cancer
Source: BJUI Compass. 2026 May 5;7(5):e70186. doi: 10.1002/bco2.70186 (PMC13143510; doi:10.1002/bco2.70186)
Supplement: Supplementary file 6 — Figure S6: Alpha diversity comparisons between favourable risk IMDC RCC patients (Red, n = 8) and intermediate plus poor risk IMDC patients (Blue, n = 21). Wilcoxon ranksum test was used. [file BCO2-7-e70186-s004.docx]

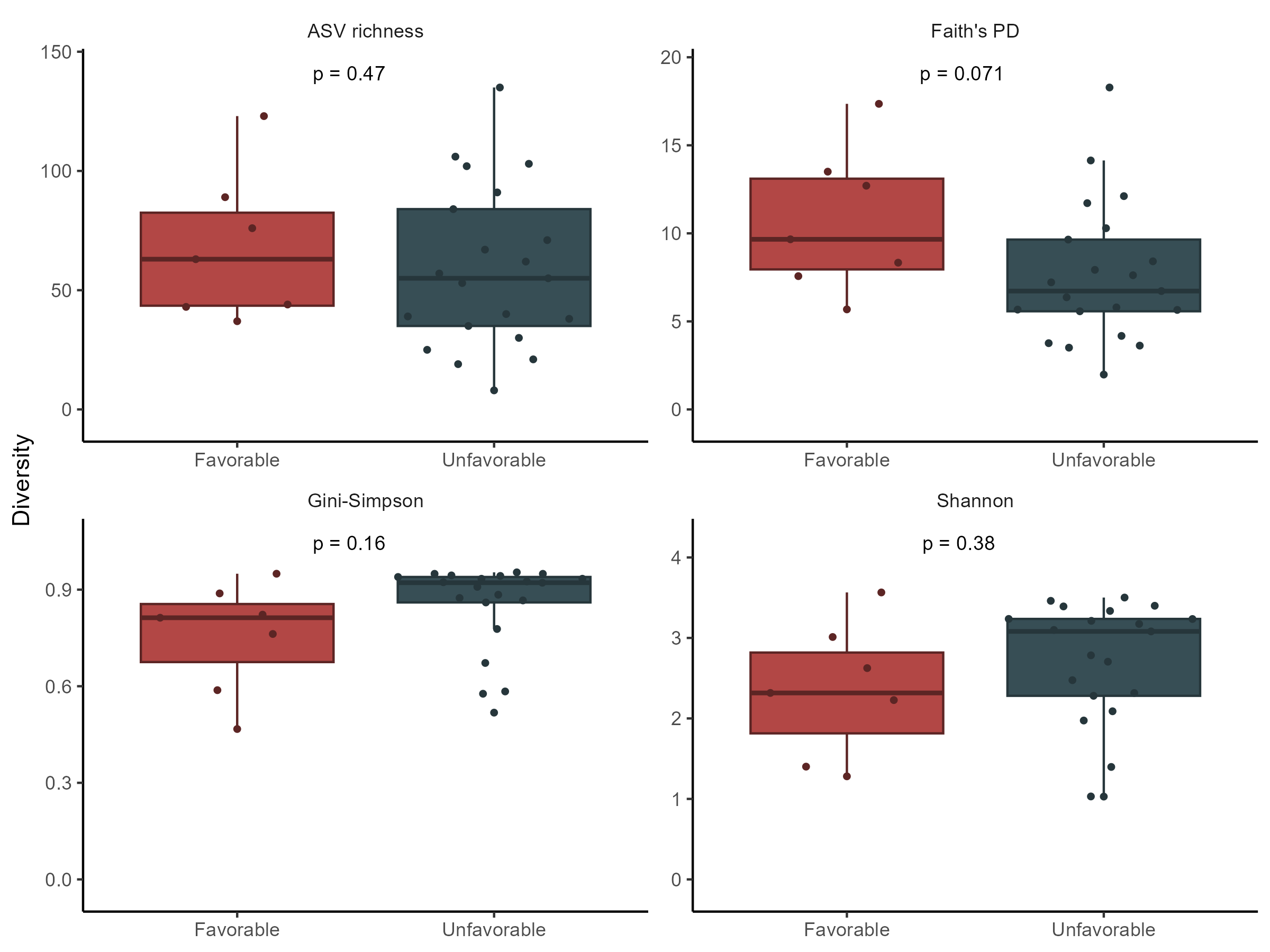


Supplemental Figure 6: Alpha diversity comparisons between favorable risk IMDC RCC patients (Red, n = 8) and intermediate plus poor risk IMDC patients (Blue, n=21). Wilcoxon ranksum test was used
