# Supplementary material for: Urinary microbiota diversity and composition in patients with advanced renal cell cancer
Source: BJUI Compass. 2026 May 5;7(5):e70186. doi: 10.1002/bco2.70186 (PMC13143510; doi:10.1002/bco2.70186)
Supplement: Supplementary file 7 — Figure S7: (A) Beta diversity comparison between favourable risk IMDC RCC patients (Red) and intermediate plus poor risk IMDC patients (Blue) using the Jaccard. (B) Bray–Curtis. (C) Nonweighted UniFrac. (D) weighted UniFrac metrics. PERMANOVA test was used. [file BCO2-7-e70186-s008.docx]

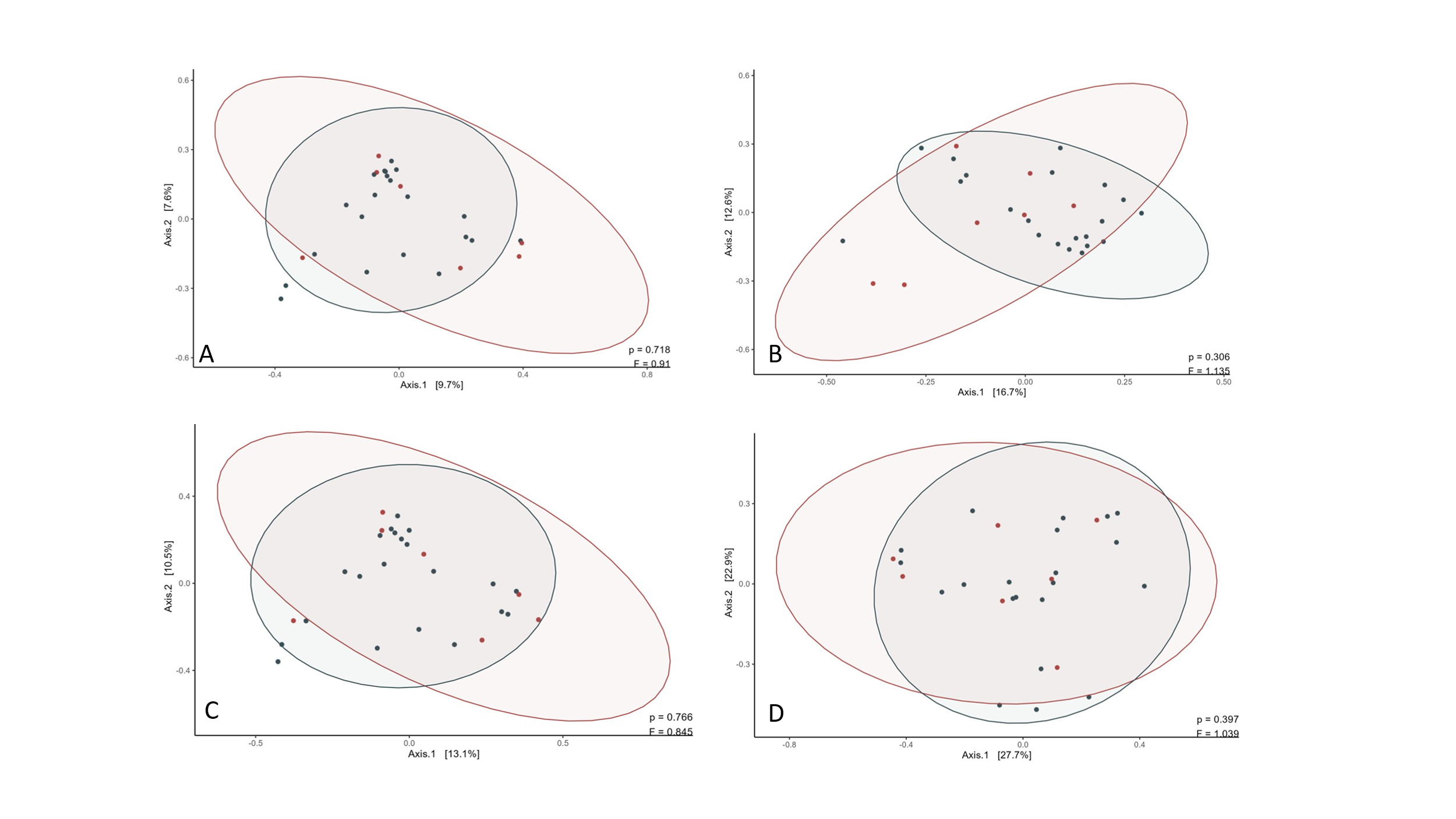


Supplemental Figure 7: A: Beta diversity comparison between favorable risk IMDC RCC patients (Red) and intermediate plus poor risk IMDC patients (Blue) using the Jaccard B: Bray-Curtis C: non-weighted UniFrac D: weighted UniFrac metrics. PERMANOVA test was used.
