# Supplementary material for: Urinary microbiota diversity and composition in patients with advanced renal cell cancer
Source: BJUI Compass. 2026 May 5;7(5):e70186. doi: 10.1002/bco2.70186 (PMC13143510; doi:10.1002/bco2.70186)
Supplement: Supplementary file 8 — Figure S8: (A) Beta diversity comparison between pretreatment RCC samples (Red) posttreatment samples (Blue) using the Jaccard. (B) Bray–Curtis. (C) weighted UniFrac metrics. PERMANOVA test was used. [file BCO2-7-e70186-s003.docx]

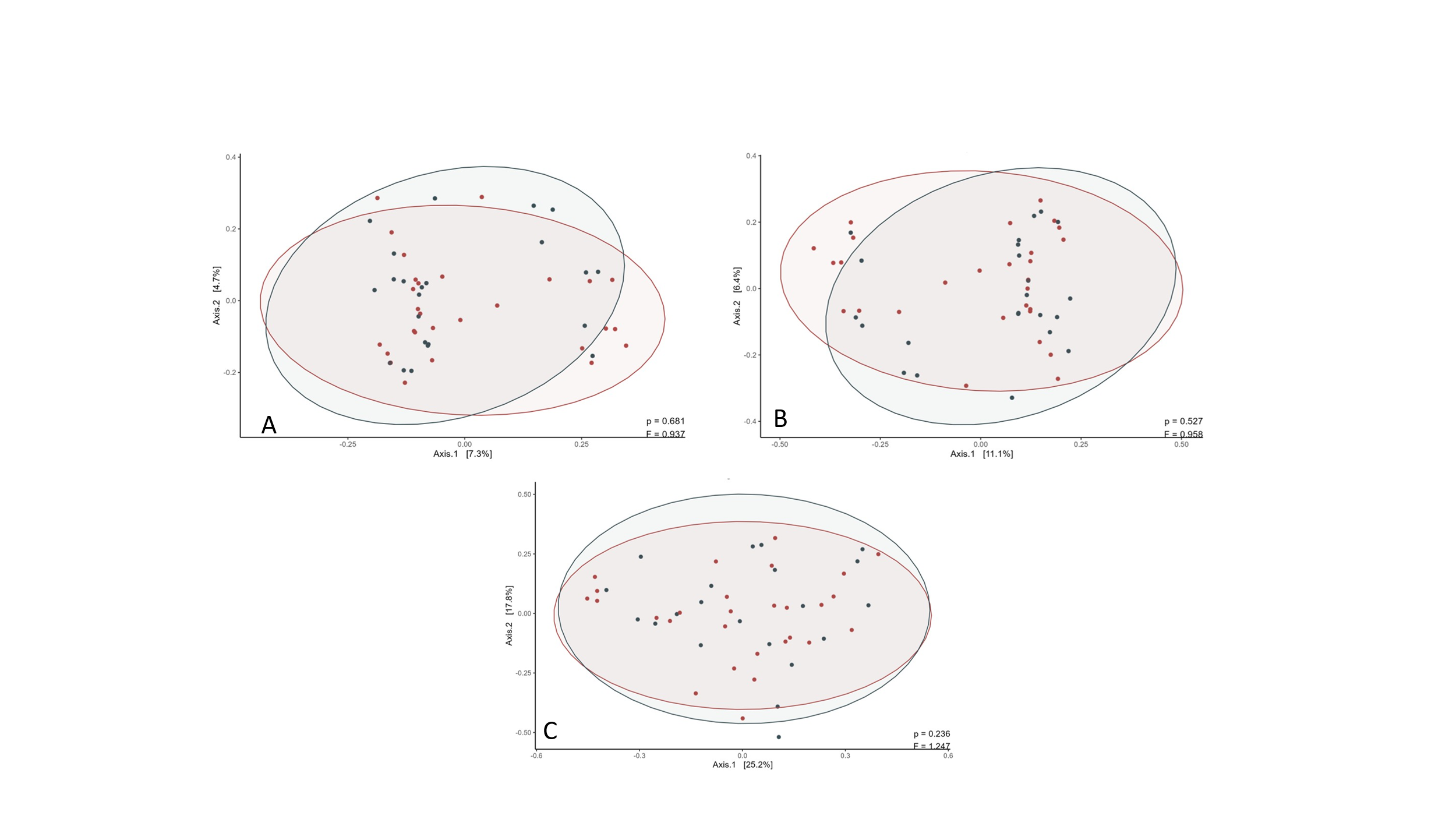


Supplemental Figure 8: A: Beta diversity comparison between pretreatment RCC samples (Red) posttreatment samples (Blue) using the Jaccard B: Bray-Curtis C: weighted UniFrac metrics. PERMANOVA test was used.
